# The Effect of Mesenchymal Stem Cell-Derived Microvesicles on Erythroid Differentiation of Umbilical Cord Blood-Derived CD34^+^ Cells

**DOI:** 10.15171/apb.2018.034

**Published:** 2018-06-19

**Authors:** Davod Pashoutan Sarvar, Mohammad Hossein Karimi, Aliakbar Movassaghpour, Parvin Akbarzadehlaleh, Sara Aqmasheh, Hamze Timari, Karim Shamsasenjan

**Affiliations:** ^1^Stem Cell Research Center, Tabriz University of Medical Sciences, Tabriz, Iran.; ^2^Transplant Research Center, Shiraz University of Medical Sciences, Shiraz, Iran.; ^3^Hematology & Blood Banking, Hematology and Oncology Research Center, Tabriz University of Medical Sciences, Tabriz, Iran.; ^4^Department of Pharmaceutical Biotechnology, Faculty of Pharmacy, Tabriz University of Medical Sciences, Tabriz, Iran.

**Keywords:** CD34^+^ cells, Mesenchymal stem cells, Microvesicles, Erythroid differentiation

## Abstract

***Purpose:*** Mesenchymal stem cells (MSCs) play an important role in the proliferation and differentiation of hematopoietic stem cells (HSCs) in the bone marrow via cell-to-cell contact, as well as secretion of cytokines and microvesicles (MVs). In this study, we investigated the effect of mesenchymal stem cell-derived microvesicles (MSC-MVs) on erythroid differentiation of umbilical cord blood-derived CD34^+^ cells.

***Methods:*** In this descriptive study, CD34^+^ cells were cultured with mixture of SCF (10 ng/ml) and rhEPO (5 U/ml) cytokines in complete IMDM medium as positive control group. Then, in MV1- and MV2-groups, microvesicles at 10 and 20 µg/ml concentration were added. After 72 hours, erythroid specific markers (CD71 and CD235a) and genes (HBG1, GATA1, FOG1 and NFE2) were assessed by flow cytometry and qRT-PCR, respectively.

***Results:*** The expression of specific markers of the erythroid lineages (CD71 and GPA) in the presence of different concentration of microvesicles were lower than that of the control group (P<0.001). Also, the expression of specific genes of the erythroid lineages (NFE2, FOG1, GATA1, and HBG1) was investigated in comparison to the internal control (GAPDH). Among all of them, HBG1 and FOG1 genes were significantly decreased to the control group (P<0.0001) but GATA1 and NFE2 gene expressions was not significant.

***Conclusion:*** The results of this study showed that MSC-MVs decrease the erythroid differentiation of umbilical cord blood-derived CD34^+^ cells. Therefore, MSC-MVs play a key role in the regulation of normal erythropoiesis.

## Introduction

Hematopoiesis is the process of mature blood cell production from hematopoietic stem cells (HSCs) in the bone marrow. Bone marrow mesenchymal stem cells (BMMSCs), as non-hematopoietic cells and main components of stromal cell niche, play a pivotal role in the regulation of normal hematopoiesis.^1,2^ MSCs support the maintenance of HSCs, as well as inhibits apoptosis, stimulates the proliferation and differentiation of these cells.^3-5^ In addition, MSCs augment of engraftment and hematopoiesis of HSCs after hematopoietic stem cell transplantation (HSCT).^6,7^ MSCs exert their roles via direct cell-to-cell, as well as secretion of various soluble mediators such as growth factors, cytokines, and extracellular vesicles (EVs).^8^ Microvesicles (MVs) are one type of the extracellular vesicles which originate from the cell membranes and have 100-1000 nm size.^9^ In addition to stem cells, microvesicles are also produced and secreted by leukocytes, platelets, dendritic cells, adipocytes, neurons, mast cells and other cells under physiological or pathological conditions.^10^ Also, MVs present in numerous body fluids and supernatant of cell cultures.^11^ As a result of their source, microvesicles derived numerous cell types contain membrane-associated proteins such as tetraspanins (e.g. CD9, CD63 and CD81), heat-shock proteins (e.g. Hspa8, Hsp60, Hsp70 and Hsp90), cytoskeletal proteins (e.g. actin, syntenin and moesin) and proteins implicated in multivesicular body synthesis (Alix and TSG101), as well as other markers related to the cells of origin including CD29, CD73 and CD44 in MSCs.^12,13^ In addition to proteins, MVs contain lipids (cholesterol, sphingomyelin, ceramide, phospholipids, and glucans), DNA, mRNA, and small non-coding RNAs (e.g. miRNAs and siRNAs).^14^

MSC-derived microvesicles (MSC-MVs) by transferring proteins and trophic mediators to adjacent or distant cells play important roles in intercellular communications.^15^ MSC-MVs changes the proliferation, differentiation and the gene expression of the target cells.^16^ Several studies have demonstrated that MSC-MVs involved in repairing of injured tissues (e.g. kidney, heart, brain, liver and bone marrow) and adjustment of immune responses.^13,17,18^ Also, previous studies proved that non-coding RNAs play a crucial function in cell proliferation, development, and differentiation.^19,20^ There are several microRNAs (e.g. miR-22, miR-24, miR-miR-144, miR-221, miR-222 and miR-451) in the microvesicles, which have been shown to play a key role in regulating the erythroid differentiation of hematopoietic stem cells.^21,22^

Nowadays, HSCs (CD34^+^) are co-transplanted with MSCs in the treatment of hematological diseases‏. On the other hand, because of MSCs have the supportive role in hematopoiesis and it is guessed that the MSC-MVs simulate the same effect of MSCs, it is necessary to investigate the effect of MSC-MVs on determining HSC fate. Therefore, in the present study, we isolated CD34^+^ cells from umbilical cord blood, as well as, microvesicles from MSCs culture *in vitro*. Finally,‏ the impact of MSC-MVs on erythroid differentiation of CD34^+^ cells were assayed.

## Materials and Methods

### Sample collection

Umbilical Cord Blood (UCB) were collected from healthy full-term normal deliveries after obtaining informed consent in the Alzahra hospital of Tabriz province. UCB samples were collected in heparinized tubes and transferred to the laboratory at 4°C‏ immiately‏.

### UCB-derived CD34^+^ cell separation and flow cytometry

Cell separation was done within 4 hours after collection. **M**ononuclear cells (MNCs) were obtained by centrifugation over Ficoll-Hypaque density gradient (1.077 g/cm^3^, GE Healthcare) and CD34^+^ cells were purified by using an immunomagnetic cell sorting (MACS) technology according to the manufacturer’s instruction (Miltenyi Biotec, Bergisch-Gladbach, Germany). As well as, purity of isolated UCB-derived CD34^+^ assessed by flow cytometry.

### UCB-derived CD34^+^ cell culture

After isolation, UCB-derived CD34^+^were cultured at a density of 1 × 10^5^ cells/ml in Iscove’s Modified Dulbecco’s Medium (IMDM) (GIBCO, Life Technologies Inc., UK) supplemented with 10% fetal bovine serum (FBS), 100 U/ml penicillin, 100 U/ml streptomycin and 0.2 mM L-glutamine at 37°C and 5% CO2 in a humidiﬁed atmosphere incubator. In order to induce cell proliferation, combination of recombinant human stem cell factor (rhSCF), thrombopoietin (TPO) and FLT3-ligand (FL) were add at 100 ng/ml final concentrations. Cell number and viability were determined by trypan blue dye exclusion method using a hemocytometer.

### Culture of MSCs and microvesicle isolations

MSCs were defreezed and cultured in complete Dulbecco's Modified Eagle Medium (DMEM) (GIBCO, Life Technologies Inc., UK) containing with 10% FBS, 100 U/ml penicillin, 100 U/ml streptomycin and 0.2 mM L-glutamine at 37°C and 5% CO2 in a humidiﬁed atmosphere incubator. At near 70% confluence, washed 3 times with PBS and incubated in serum-free media. After 24 hours, concentrated culture medium (CCM) was collected and centrifuged at 300 ×g for 10 min to remove nonadherent cells and cell debris. The supernatant was collected and centrifuged at 16,000 ×g for 1 hours at 4°C. Supernatant was removed, and then the pelleted MVs were washed with PBS and centrifuged twice at 100,000 ×g for 1 h at 4°C (Ultracentrifuge, Beckman, USA).^13,23^ Finally, the supernatant was removed, and the pelleted MVs were suspended with PBS and stored at -80°C for subsequent experiments. The protein quantity of MVs was determined by using Bradford assay (Pierce, Rockford, IL, USA). In addition, the size of MSC-MVs were determined using Dynamic Light Scattering (DLS) (Zetasizer NanoZS, Malvern Instruments, UK).

### Treatment of UCB-derived CD34^+^cells with MSC-MVs

In order to investigate the effect of MSC-MVs on erythroid differentiation of UCB-derived CD34^+^ cells, CD34^+^ cells (1×10^5^/ml) were incubated in IMDM supplemented with 10% FBS for 72 hours under three culture conditions: (1) The control group: Recombinant cytokines including mixture of rhSCF (10 ng/ml) and rhEPO (5 U/ml), (2) The MV_1_ group: Recombinant cytokines and 10 µg/ml concentration of microvesicles, (3) The MV_2_ group: Recombinant cytokines and 20 µg/ml concentration of microvesicles.

### Assessment of erythroid differentiation by flow cytometry

In order to determine erythroid differentiation of UCB-derived CD34^+^, after 72 hours the cells (1×10^5^/ml) related to each group were gathered for flow cytometric analysis. Initially, cells were washed and suspended in PBS and labeled on ice (4°C) with anti-CD71-PE and anti-CD235a-FITC (DakoCytomation, Glostrup, Denmark) for 30 min. Then cells were rewashed and resuspended in PBS (as sheath fluid) for flowcytometric analysis. Data were acquired by FACSCalibur equipped with the CellQuest software package (BD Biosciences), and finally analyzed by Flowing software (Turk University, Finland).

### RNA isolation and qRT-PCR

To investigation of specific gene expressions in erythroid lineage differentiation, we studied *HBG1*, *GATA1*, *FOG1* and *NFE2* genes after 72 hours incubation periods. Initially, total cellular RNA were isolated from each group cells by QIAzol lysis reagent (QIAGEN, USA) based on the manufacturer’s instructions. In addition, RNA concentration and quality was assayed by the spectrophotometric absorbance ratio at 260/280 nm (Picodrop, UK). cDNA synthesis were performed using BioRT cDNA first strand synthesis Kit protocol (Bioer, Japan) according to the manufacturer's protocol. Erythroid specific gene (*HBG1*, *GATA1*, *FOG1* and *NFE2*) expressions were assayed by RT-PCR. In this process of amplification, we added 5 µl of 2X qPCR/RTD-PCR Master mix E4 (SYBR Green AB kit) to 1 µl forward primer, 1 µl reverse primer (Metabion, Germany), 1 µl cDNA, and 2 µl ddH2O. Reactions were performed with the Applied Biosystems Step One Real Time - PCR System (Applied Biosystem, USA) under 95°C for 10 minutes, next 40 cycles as follows: 95 °C for 15 seconds and 60 °C for 60 seconds. The housekeeping gene *GAPDH* in each sample was used as an internal control. The relative gene expression of genes were calculated using the 2^-ΔΔCt^ method. The pairs of erythroid gene-specific primers were used are described in Table 1.

**Table 1:** Primers for Real Time – PCR

**Gene**	**Primer**
HBG1	Forward: 5′-GGAAGATGCTGGAGGAGAAACC-3′ Reverse: 5′-GTCAGCACCTTCTTGCCATGTG-3′
GATA1	Forward: 5′-CACGACACTGTGGCGGAGAAAT-3′ Reverse: 5′-TTCCAGATGCCTTGCGGTTTCG-3′
NFE2	Forward: 5′-GGAGAGATGGAACTGACTTGGC-3′ Reverse: 5′-GAATCTGGGTGGATTGAGCAGG-3′
FOG-1	Forward: 5′-TTCGTGTGCCTGATCTGCCTGT-3′ Reverse: 5′-GTTGGTGACCAAGTGGCTGTAG-3′
GAPDH	Forward: 5′-ACCCATCACCATCTTCCAGGAG-3′ Reverse: 5′-GAAGGGGCGGAGATGATGAC-3′

### Statistical Analysis

All experiments were carried out in triplicate. The obtained data from the study were presented as the mean ± SD and were analyzed by GraphPad Prism v5.00 (GraphPad Software, San Diego, CA, USA). Statistical analysis for multiple comparisons was performed by using one-way ANOVA, as well as student’s t-test for single comparisons. A p value < 0.05 was considered to be statistically significant for all experiments.

## Results

### Flow cytometry of UCB-derived CD34^+^ cells

**Figure 1:**
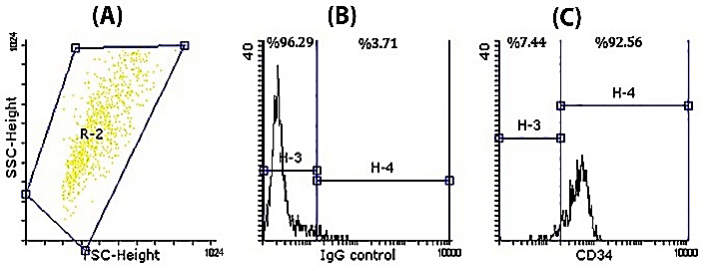
Flow cytometric analysis of isolated UCB-CD34^+^ cells. **A.** Forward versus side scatter (FSC/SSC) dot plot with the gated CD34^+^ cells in region R-2. **B.** Negative isotype control (mouse IgG1). **C.** CD34^+^ cell populations in region H-4 (purity of isolated UCB-CD34^+^ cells were %92.56).

Purity of UCB-derived CD34^+^cells isolated by MidiMACS using flow cytometry were 92.56% (Figure 1). In addition, viability of thesecells were 93±2% by trypan blue dye exclusion method.

### Characterization of MSC-MVs

**Figure 2:**
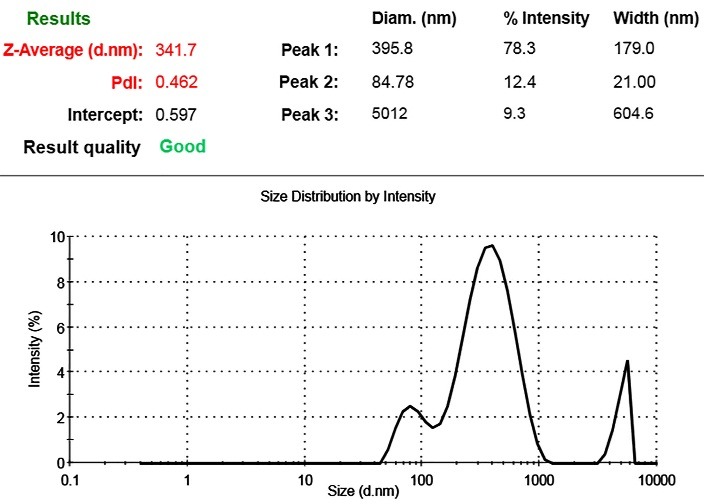
Dynamic light scattering (DLS) analysis for the size distribution of MSC-MVs. The average size of isolated MSC-MVs were 341 nm.

The size of MSC-MVs were determined by Dynamic Light Scattering (DLS). The results showed that isolated MSC-MVs have the mean size of 341 nm (Figure 2). In addition to size, protein concentration of MSC-MVs were calculated by using Bradford assay. The results showed that the average concentration of MSC-MVs was 171 µg/ml.

### The effects of MSC-MVs on erythroid differentiation of UCB-derived CD34^+^cells

#### Flowcytometric evaluation of CD71 and CD235a

**Figure 3:**
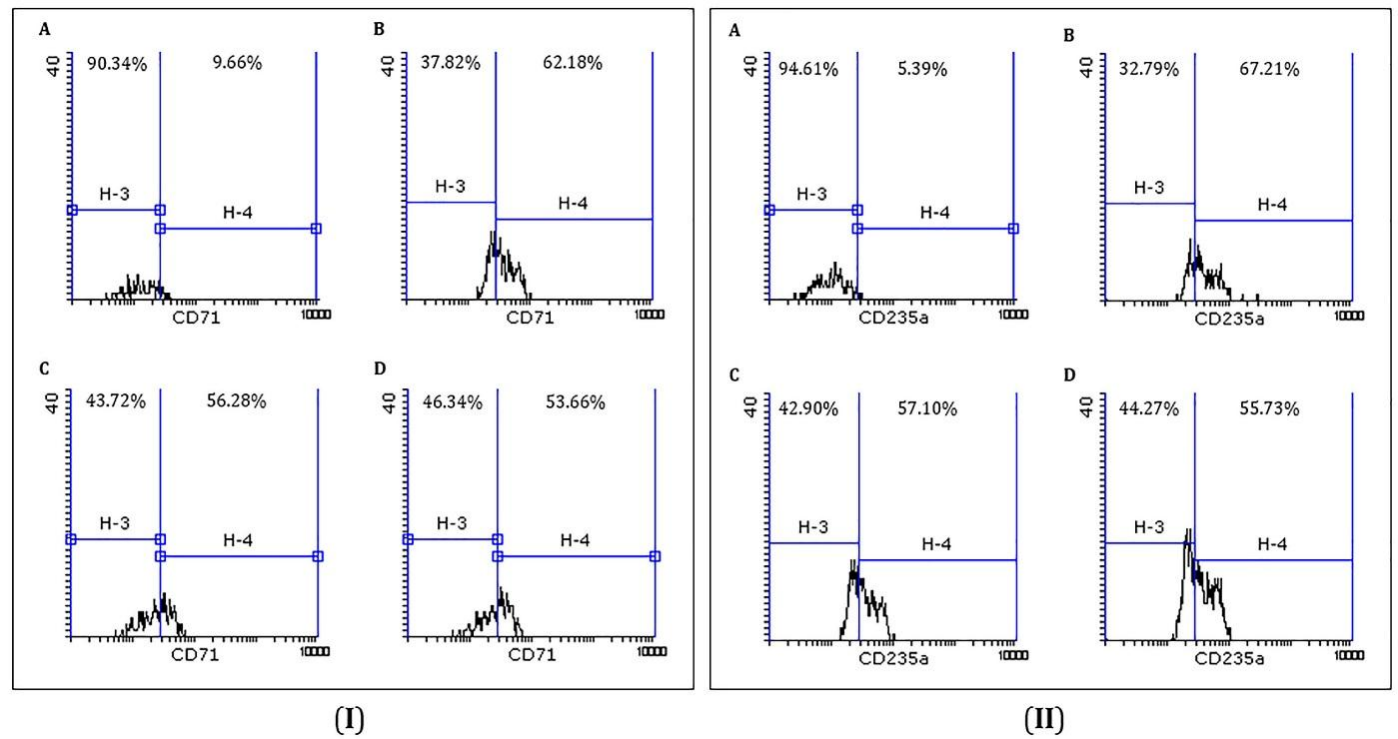
Representative flow cytometric analysis of CD71 (**I**) and CD235a (**II**) expressions. **A.** CD34^+^ cells. **B,** Upregulation of CD71 and CD235a in presence of recombinant cytokines (rhEPO and rhSCF). **C** and** D.** Downregulation‏ of erythroid differentiation of CD34^+^ cells with addition of 10 and 20 µg/ml concentration of microvesicles, respectively (p value < 0.001).

Flowcytometric analysis showed that an increase in the percentage of CD71 and CD235a marker expressions in presence of cytokine mixture containing rhSCF and rhEPO in comparison to primary UCB-derived CD34^+^cells. This results indicate that erythroid lineage commitment and differentiation were occurred but with addition of different concentration of microvesicles in MV_1_- and MV_2_-groups, erythroid differentiation of CD34^+^ cells were significantly reduced (p value < 0.001) (Figure 3). Expression of CD71 and CD235a related to MV_1_- and MV_2_-groups in comparison to each other were not significant.

#### Gene expressions analysis of HBG1, FOG1, GATA1 and NF-E2

**Figure 4:**
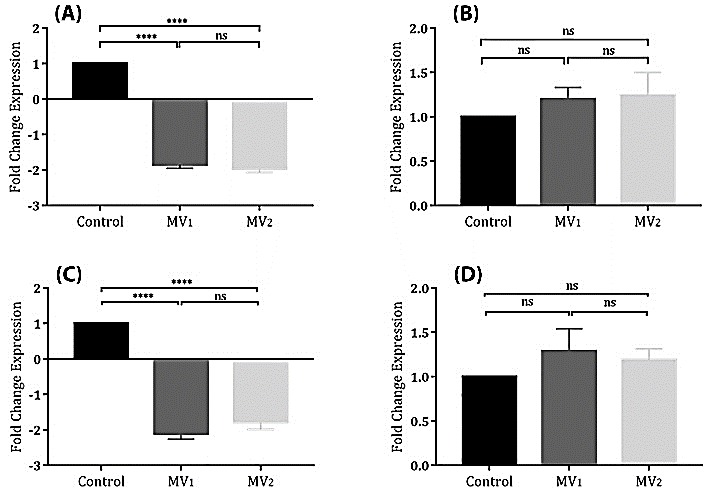
The relative expression of HBG1 (**A**), GATA1 (**B**), FOG1 (**C**), and NFE2 (**D**) genes were investigated by Real Time-PCR in three differentiation conditions after 72 hours. GAPDH was used as internal control. HBG1 and FOG1 genes were significantly down-regulated in MV_1_- and MV_2_-treated groups‏ in comparison to control group. Upregulation of GATA1 and NFE2 genes were not significant to the control group. ****; Denotes p value <0.0001. ns; non-significant.

In MV_1_- and MV_2_-groups in comparison to control group, real time-PCR analysis showed a marked decrease in *HBG1* and *FOG1* gene expressions (p value < 0.0001); no significant increase in *GATA1* and *NFE2* gene expressions. In all gene-related conditions, there was no significant changes between MV_1_- and MV_2_-groups for genes (Figure 4).

## Discussion

MSC-MVs play an important role in determining HSC fate because they have been enriched with a wide range of‏ growth factors and small non-coding RNAs including miRNAs and siRNAs.^22,24^ Therefore, the main aim of our current research was to the assessment of the‏ impacts of MSC-MVs on the erythroid differentiation of UCB-derived CD34^+^cells in an induced conditions. The results of this study showed that MSC-MVs could reduce erythroid differentiation of selected UCB-derived CD34^+^cells *in vitro*.

A study reported that MSCs induce HSCs viability and proliferation, as well as have a supportive effect on myeloid lineage differentiation instead of erythroid differentiation. In addition, among all component of studied extracellular matrix (ECM) proteins, only laminin and ECM gel had a supportive effects on erythroid differentiation.^25^ Saleh et al. demonstrated that MSCs have an inhibitory effect on the erythroid differentiation of K562 cell lines.^26^ Our results were in accordance with two described studies, which both showed a decreased effect of MSCs on HSCs erythroid differentiation but from the point of paracrine effects between MSCs and HSCs.

Many studies proved that MSC-MVs involved in tissue regeneration specially bone marrow.^15,16,27^ For the first time in 2016, Xie et al. demonstrated that MSC-MVs increase the proliferation and colonogenesis of CD34^+^ cells derived from the umbilical cord blood *in vitro*. As well as, the addition of MSC-MVs into the MSCs and CD34^+^ cells co-culture system induces the proliferation of primary CD34^+^ cells.^28^ Additionally, another study showed that BMMSC-derived vesicles restoring radiation-induced bone marrow damage by increasing HSC proliferations and inhibition of DNA damages.^29^ Also, vesicles derived from MSCs prevent HSCs apoptosis by increasing CXCR-4 and chemokine expressions.^30^

According to above studies, vesicles derived from MSCs play an important role in the specification of HSC fate such as proliferation and apoptosis, probably differentiation. Hence, we survived the effects of MSC-MVs on the erythroid differentiation of UCB-derived CD34^+^cells.

Both CD71 (transferrin receptor) and CD235a (glycophorin A) are two specific markers that express during erythroid maturation.^31^ In our study, the percentage of CD71^+^/CD235a^+^ in the presence of microvesicles were decreased which indicating a reduction of erythroid differentiation. As well as, we studied the erythroid specific genes including *HBG1*, *FOG1*, *GATA1*, and *NFE2* genes by using qRT-PCR. Friend of *GATA-1* (*FOG1*) is a nuclear protein that binds to transcription factor *GATA-1* and play an important role in early erythropoiesis.^32^ We showed that MSC-MVs inhibited expressions of *FOG1* gene. Human fetal γ-globin genes including *HBG1* (A_γ_) and *HBG2* (G_γ_) which express in early and late erythroid maturation. γ-Globin in combination with α-globin form the HbF tetramer.^33^ Based on our results, downregulation of *HBG1* during erythropoiesis can leads to inhibition of erythroid differentiation in the presence of microvesicles. Other erythroid specific genes show no significant expressions. Therefore, further researches is necessary to the assessment of these genes.

Our results demonstrated that MSC-MVs can lead to downregulation of *HBG-1* and *FOG-1*, as well as, specific erythroid lineage surface markers such as CD71 and CD235a expressions. These findings show that MSC-MVs can suppress the erythroid differentiation of UCB-derived CD34^+^cells.

## Conclusion

Many studies assayed interactions between MSCs and HSCs in co-culture systems. These studies have proved that MSCs support hematopoiesis via direct cell-to-cell contact and secretion of paracrine mediators. In current study, we demonstrated that MSC-MVs suppress erythroid differentiation of UCB-derived CD34^+^ cells. Therefore, the inhibitory effects of MSC-MVs on normal erythropoiesis should be considered when these vesicles were applied in cell-free therapy. Additionally, the accurate mechanism underlying the erythropoiesis-suppressing effect of MSC-MVs remains unknown. Further studies should be done for determine the cellular and molecular mechanisms involved in the effects of MSC-MVs on differentiation of UCB-derived CD34^+^ cells.

## Acknowledgments

Authors would like to special thank the Stem Cell Research Centre of Tabriz University of Medical Sciences. We would also like to appreciate the Tabriz Blood Transfusion Headquarter to provide laboratory facilities.

## Ethical Issues

Not applicable

## Conflict of Interest

The authors report no conflicts of interest.

## References

[1] Bernardo ME, Locatelli F (2016). Mesenchymal stromal cells in hematopoietic stem cell transplantation. Methods Mol Biol.

[2] Spees JL, Lee RH, Gregory CA (2016). Mechanisms of mesenchymal stem/stromal cell function. Stem Cell Res Ther.

[3] Rodriguez-Pardo VM, Vernot JP (2013). Mesenchymal stem cells promote a primitive phenotype CD34^+^c-kit+ in human cord blood-derived hematopoietic stem cells during ex vivo expansion. Cell Mol Biol Lett.

[4] Batsali AK, Kastrinaki MC, Papadaki HA, Pontikoglou C (2013). Mesenchymal stem cells derived from wharton's jelly of the umbilical cord: Biological properties and emerging clinical applications. Curr Stem Cell Res Ther.

[5] Jing D, Fonseca AV, Alakel N, Fierro FA, Muller K, Bornhauser M (2010). Hematopoietic stem cells in co-culture with mesenchymal stromal cells--modeling the niche compartments in vitro. Haematologica.

[6] Carrancio S, Blanco B, Romo C, Muntion S, Lopez-Holgado N, Blanco JF (2011). Bone marrow mesenchymal stem cells for improving hematopoietic function: An in vitro and in vivo model. Part 2: Effect on bone marrow microenvironment. PLoS One.

[7] Noort WA, Kruisselbrink AB, in't Anker PS, Kruger M, van Bezooijen RL, de Paus RA (2002). Mesenchymal stem cells promote engraftment of human umbilical cord blood-derived cd34(+) cells in nod/scid mice. Exp Hematol.

[8] Bruno S, Collino F, Tetta C, Camussi G (2013). Dissecting paracrine effectors for mesenchymal stem cells. Adv Biochem Eng Biotechnol.

[9] Szatanek R, Baj-Krzyworzeka M, Zimoch J, Lekka M, Siedlar M, Baran J. The methods of choice for extracellular vesicles (evs) characterization. Int J Mol Sci 2017;18(6). doi: 10.3390/ijms18061153 10.3390/ijms18061153 PMC5485977 28555055

[10] Rani S, Ryan AE, Griffin MD, Ritter T (2015). Mesenchymal stem cell-derived extracellular vesicles: Toward cell-free therapeutic applications. Mol Ther.

[11] Thery C, Ostrowski M, Segura E (2009). Membrane vesicles as conveyors of immune responses. Nat Rev Immunol.

[12] Raposo G, Stoorvogel W (2013). Extracellular vesicles: Exosomes, microvesicles, and friends. J Cell Biol.

[13] Bruno S, Grange C, Deregibus MC, Calogero RA, Saviozzi S, Collino F (2009). Mesenchymal stem cell-derived microvesicles protect against acute tubular injury. J Am Soc Nephrol.

[14] Than UTT, Guanzon D, Leavesley D, Parker T. Association of extracellular membrane vesicles with cutaneous wound healing. Int J Mol Sci 2017;18(5). doi: 10.3390/ijms18050956 10.3390/ijms18050956 PMC5454869 28468315

[15] Biancone L, Bruno S, Deregibus MC, Tetta C, Camussi G (2012). Therapeutic potential of mesenchymal stem cell-derived microvesicles. Nephrol Dial Transplant.

[16] Sabin K, Kikyo N (2014). Microvesicles as mediators of tissue regeneration. Transl Res.

[17] Borger V, Bremer M, Ferrer-Tur R, Gockeln L, Stambouli O, Becic A, et al. Mesenchymal stem/stromal cell-derived extracellular vesicles and their potential as novel immunomodulatory therapeutic agents. Int J Mol Sci 2017;18(7). doi: 10.3390/ijms18071450 10.3390/ijms18071450 PMC5535941 28684664

[18] Chen B, Li Q, Zhao B, Wang Y (2017). Stem cell-derived extracellular vesicles as a novel potential therapeutic tool for tissue repair. Stem Cells Transl Med.

[19] Hwang HW, Mendell JT (2007). Micrornas in cell proliferation, cell death, and tumorigenesis. Br J Cancer.

[20] Huleihel L, Scarritt ME, Badylak SF (2017). The influence of extracellular rna on cell behavior in health, disease and regeneration. Curr Pathobiol Rep.

[21] Chen TS, Lai RC, Lee MM, Choo AB, Lee CN, Lim SK (2010). Mesenchymal stem cell secretes microparticles enriched in pre-micrornas. Nucleic Acids Res.

[22] Lazare SS, Wojtowicz EE, Bystrykh LV, de Haan G (2014). Micrornas in hematopoiesis. Exp Cell Res.

[23] Momen-Heravi F, Balaj L, Alian S, Mantel PY, Halleck AE, Trachtenberg AJ (2013). Current methods for the isolation of extracellular vesicles. Biological chemistry.

[24] Armstrong JP, Holme MN, Stevens MM (2017). Re-engineering extracellular vesicles as smart nanoscale therapeutics. ACS Nano.

[25] Lazar-Karsten P, Dorn I, Meyer G, Lindner U, Driller B, Schlenke P (2011). The influence of extracellular matrix proteins and mesenchymal stem cells on erythropoietic cell maturation. Vox Sang.

[26] Saleh M, Shamsasanjan K, Movassaghpour AA, Akbarzadehlaleh P, Molaeipour Z (2017). Inhibitory effect of mesenchymal stem cell co-culture on erythroid differentiation of k562 cells compared to the control group. Cell J.

[27] Kode JA, Mukherjee S, Joglekar MV, Hardikar AA (2009). Mesenchymal stem cells: Immunobiology and role in immunomodulation and tissue regeneration. Cytotherapy.

[28] Xie H, Sun L, Zhang L, Liu T, Chen L, Zhao A (2016). Mesenchymal stem cell-derived microvesicles support ex vivo expansion of cord blood-derived cd34(+) cells. Stem Cells Int.

[29] Wen S, Dooner M, Cheng Y, Papa E, Del Tatto M, Pereira M (2016). Mesenchymal stromal cell-derived extracellular vesicles rescue radiation damage to murine marrow hematopoietic cells. Leukemia.

[30] De Luca L, Trino S, Laurenzana I, Lamorte D, Caivano A, Del Vecchio L, et al. Mesenchymal stem cell derived extracellular vesicles: A role in hematopoietic transplantation? Int J Mol Sci 2017;18(5). doi: 10.3390/ijms18051022 10.3390/ijms18051022 PMC5454935 28486431

[31] Fajtova M, Kovarikova A, Svec P, Kankuri E, Sedlak J (2013). Immunophenotypic profile of nucleated erythroid progenitors during maturation in regenerating bone marrow. Leuk Lymphoma.

[32] Amigo JD, Ackermann GE, Cope JJ, Yu M, Cooney JD, Ma D (2009). The role and regulation of friend of gata-1 (fog-1) during blood development in the zebrafish. Blood.

[33] Sankaran VG, Xu J, Orkin SH (2010). Advances in the understanding of haemoglobin switching. Br J Haematol.

